# VARIATION IN STATE ENFORCEMENT REGULATIONS REGARDING FINANCIAL PENALTIES AGAINST ASSISTED LIVING FACILITIES

**DOI:** 10.1093/geroni/igac059.2828

**Published:** 2022-12-20

**Authors:** Jacy Weems, Kali Thomas

**Affiliations:** Brown University School of Public Health, Providence, Rhode Island, United States; Brown University, Providence, Rhode Island, United States

## Abstract

Provider behavior is known to be driven by market forces, including costs. Financial penalties are the primary enforcement remedy available to state agencies in deterring substandard practices in assisted living (AL) communities. States develop licensing and enforcement requirements for AL communities, and regulations vary by license. Using state regulations and statutes sourced for AL license types from Nexis Uni, we used key phrases to extract information from all 50 states and D.C. to characterize how state agencies calculate and assess financial penalties for licensed AL community violations. Fines are assessed for each day of substandard behavior or as a one-time amount; within the nursing home sector, per-day fines tend to be higher than per-instance fines as they accumulate. We found that of the states analyzed so far (n=25), ten states (40%) may impose either per-day or per-instance fines, nine (36%) can impose per-instance fines only, three (12%) may impose per-day fines only, and three (12%) incorporated facility size in assessing fine amounts. One state had no policies concerning AL fine enforcement. The median per-instance maximum fine amount is $2,000 [IQR $500-$5,000], while the median maximum per-day fine is $500 per day [IQR $150-$2,000]. Eleven states (44%) utilize tiered amount categories based on violation scope and severity. Agencies also consider characteristics such as community size and past performance when assessing fine amounts. Inconsistent penalties across states illuminate the need to assess the impact of current regulations, identify best enforcement practices to improve overall compliance, and share best practices between enforcement agencies.

